# Radiofrequency Catheter Ablation Of Atrioventricular Nodal Reentrant Tachycardia Associated With Anomalous Drainage Of Both Superior Vena Cava Into Coronary Sinus

**Published:** 2009-09-01

**Authors:** Rakesh Yadav, Sharad Chandra, Nitish Naik, Rajnish Juneja

**Affiliations:** Department Of Cardiology, Cardiothoracic Sciences Centre, All India Institute of Medical Sciences, Ansari Nagar, New Delhi - 110029, India

**Keywords:** Atrioventricular nodal re-entrant tachycardia, abnormal superior vena cava drainage, coronary sinus, ablation

## Abstract

Total upper body drainage via left superior vena cava into coronary sinus (i.e. absent right superior vena cava) is a rare anomaly and distorts the anatomy of coronary sinus and triangle of Koch. Herewith we are reporting the first report of ablation in a patient with superior vena cava draining into coronary sinus totally. This patient with atrioventricular nodal re-entrant tachycardia, baseline left bundle branch block, transient complete heart block during electrophysiological study and total upper body venous drainage into coronary sinus had successful slow pathway ablation using anatomical approach.

## Introduction

Anatomical approach to atrioventricular nodal re-entrant tachycardia (AVNRT) ablation based on the triangle of Koch is the standard technique now, with a success rate close to 99%. Anatomy of the coronary sinus is important to guide anatomy guided ablation. Left superior vena cava (LSVC) draining into coronary sinus is not an uncommon anomaly that enlarges the coronary sinus [1]. The enlargement of the coronary sinus distorts the anatomy of triangle of Koch, making slow pathway ablation difficult [2-4]. Total upper body drainage via left superior vena cava into coronary sinus (i.e. absent right superior vena cava) is an even rare anomaly and it further distorts the anatomy of coronary sinus and triangle of Koch [1]. Herewith we are reporting the first ever reported case of AVNRT with baseline left bundle branch block who had transient complete heart block during electrophysiological study and who also had total upper body venous drainage into coronary sinus.

## Case Report

**Figure 1:**
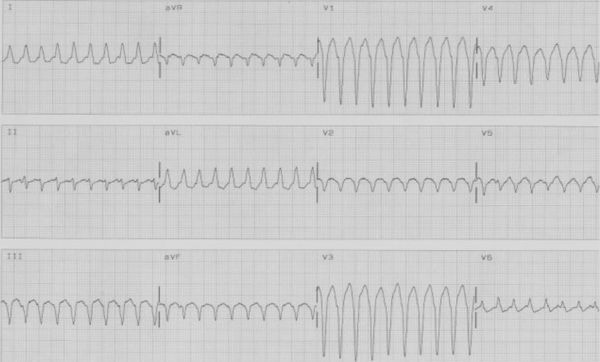
12 lead ECG of tachycardia showing heart rate of 210/minute and LBBB   with left axis morphology.

**Figure 2:**
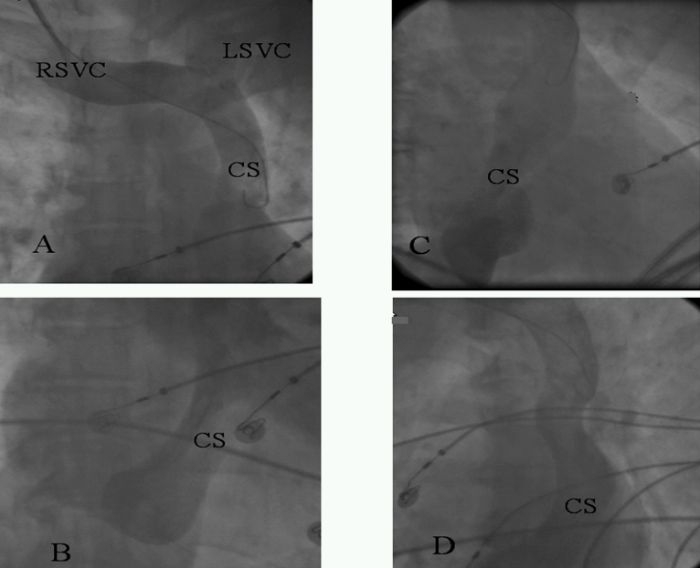
Angiogram of coronary sinus from the sheath placed into right internal jugular vein showing both right and left superior vena cava draining into dilated coronary sinus. (A and B - Left anterior oblique view,    C - right anterior oblique view, D - anterio-posterior view.)  CS - coronary sinus, RSVC - right superior vena cava, LSVC - left superior vena cava

**Figure 3:**
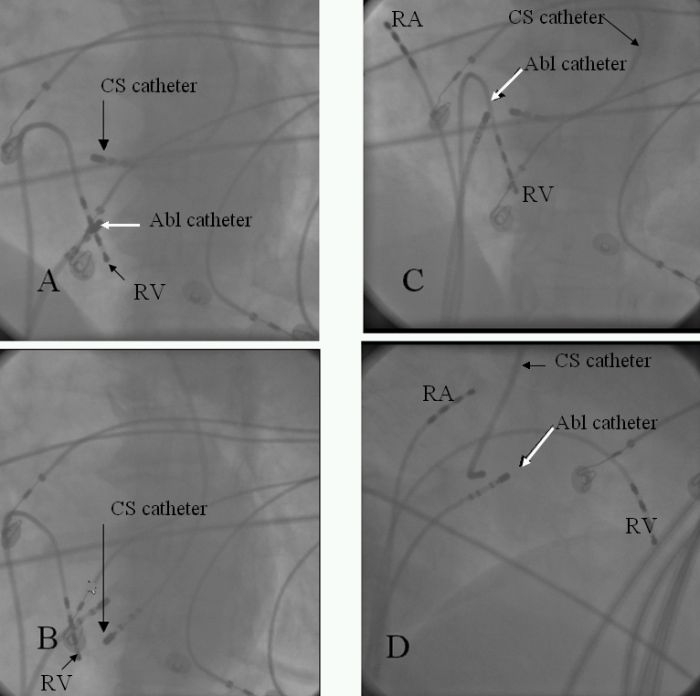
Cine-angiogram of the catheter placement. CS - coronary sinus, RV - right ventricle, RA - right atrium, abl - ablation.   Black arrow showing CS catheter tip at the upper lip (A- left anterior oblique view) and lower lip (B - left anterior oblique view) of the dilated CS ostium. White arrow showing tip of the ablation catheter at the site of successful ablation (C- left anterior oblique view, D - right anterior oblique view)

**Figure 4:**
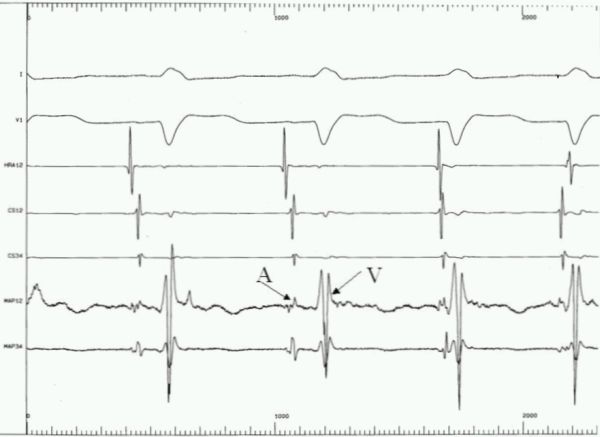
Intracardiac electrogram (speed - 100 mm/sec) at the site of successful ablation.  1st and 2nd beats are sinus &  3rd and 4th beats are junctional. HRA12 - High right atrial electrogram, CS12 &  CS34 - coronary sinus catheter electrogram, MAP12 &  MAP34 - bipolar electrogram of the mapping catheter, A- bipolar atrial signals, V - bipolar ventricular signals.

A 49 years old female presented with history of recurrent palpitations for last 10 years. During last year, she had 5 episodes of palpitations which required hospitalizations. The tachyardia was repeatedly terminated by intravenous adenosine and verapamil. She was nondiabetic and nonhypertensive. General physical and cardiovascular examination were normal. Her baseline electrocardiogram (ECG) showed left bundle branch block with normal PR interval. The electrocardiogram  of tachycardia revealed similar morphology, with the heart rate of 210 beats/min. (Figure 1). Her transthoracic echocardiogram (TTE) did not show any evidence of structural heart disease. An electrophysiological study was planned with a presumptive diagnosis of AVNRT. Catheter manipulation to obtain  His bundle electrogram led to complete heart block because of trauma to right bundle that required temporary right ventricular pacing. No tachycardia could be induced during complete heart block and there was no evidence of accessory pathway. Complete heart block persisted for 3 hours during which she required temporary ventricular pacing support. Patient was discharged after 48 hours  with a plan to restudy on recurrence. Within 15 days, she had 3 episode of tachycardia all terminated with intravenous adenosine. After the recurrence of episodes, she was taken up for a restudy with a plan of avoiding  His bundle / Right bundle injury and as a part of the same plan to avoid His recording coronary sinus catheter was put from the right internal jugular vein. Three 7 F sheaths were put into right femoral vein and one 6F sheath was put into right internal jugular vein. One catheter was put into high right atrium and another into right ventricular apex carefully avoiding trauma to His bundle. From the internal jugular vein, since the  catheter did not follow a normal course, angiogram  was done that showed  absent right superior vena cava and whole of the jugular system draining into hugely dilated coronary sinus which was missed on transthoracic echocardiogram (Figure 2). At that point of time, when we found this venous anomaly the risk of ablation was again discussed with the family and the patient, explaining them the risk of development of complete heart block and need of permanent pacemaker which would be difficult in this patient. Deflectable catheter was then used to clearly define the upper and lower lip of coronary sinus to guide the ablation (Figure 3). A 7F 4 mm tip steerable ablation catheter was positioned in right and left anterior oblique views. Tachycardia was induced and diagnosis of AVNRT was confirmed by standard protocol. The ablation was performed starting from the inferior lip of coronary sinus and gradually moving anteriorly towards the upper limb of coronary sinus. Intracardiac bipolar electrogram did not show any slow pathway potential near the coronary sinus ostium and at the site of successful ablation (Figure 4). Just at the upper limb of coronary sinus good junctional rhythm was noted (Figure 4). A complete lesion at 25-30 watts, temperature of 50ºC for 60 seconds was given. No VA or AV block was observed. Post ablation testing revealed absence of dual physiology and noninducable AVNRT. The patient was discharged on next day and till last follow up after 6 months she was asymptomatic.

## Discussion

The approach for the ablation of slow pathway in patients with anomalous drainage of superior vena cava into coronary sinus is not clear because of distorted triangle of Koch due to dilated coronary sinus [2-5]. This is, with best of our knowledge, the first ever case report of ablation of AVNRT in a patient with both superior vena cava draining into coronary sinus that makes the coronary sinus even more dilated when compared to those with only left superior vena cava draining into coronary sinus. Further more this patient had baseline left bundle branch block that led to complete heart block even with catheter manipulation to get His bundle electrogram during electrophysiological study. Permanent heart block in such a situation would be difficult problem as lead positioning would be difficult. Complete heart block is a major complication of radiofrequency ablation for treatment of supraventricular tachycardia. Complete heart block may occur early or late after radiofrequency energy application and usually it is transient. The incidence of complete heart block is around 1.3% during AVNRT ablation  [6]. Okishige et al [2] in the series of 3 patients of PLSVC draining into coronary sinus reported successful ablation of slow pathway into 2 cases, which was guided by slow pathway potential at coronary sinus ostium. They suggested that in these patients there might be atypical course of slow pathway because of distorted coronary sinus. Sakabe et al [4] found no recordable slow pathway potential any where into the coronary sinus and chose anatomical approach for ablation at posteroinferior region of triangle of Koch. Ernst S et al [5] also described anatomical approach but using the novel magnetic navigation system Niobe. Similarly an abbreviated combined anatomical and electrogram-guided approach for selective ablation of the slow pathway was found to be  a safe and curative therapeutic approach in children with atrioventricular nodal reentrant tachycardia [7]. We had also chosen the anatomical approach. In our case putting coronary sinus catheter from superior vena cava helped us during ablation, as it was easy to delineate upper and lower lip of coronary sinus by deflecting the tip of catheter into coronary sinus.
